# Improved soil biological health increases corn grain yield in N fertilized systems across the Corn Belt

**DOI:** 10.1038/s41598-020-60987-3

**Published:** 2020-03-03

**Authors:** Jordon Wade, Steve W. Culman, Jessica A. R. Logan, Hanna Poffenbarger, M. Scott Demyan, John H. Grove, Antonio P. Mallarino, Joshua M. McGrath, Matthew Ruark, Jaimie R. West

**Affiliations:** 1 School of Environment & Natural Resources, The Ohio State University, Ohio, USA; 2 Department of Crop Sciences, University of Illinois, Urbana-Champaign, Illinois, USA; 3 College of Education and Human Ecology, The Ohio State University, Ohio, USA; 4 Department of Plant and Soil Sciences, University of Kentucky, Kentucky, USA; 5 Department of Agronomy, Iowa State University, Iowa, USA; 6 Department of Soil Science, University of Wisconsin — Madison, Wisconsin, USA

**Keywords:** Biogeochemistry, Environmental sciences

## Abstract

Nitrogenous fertilizers have nearly doubled global grain yields, but have also increased losses of reactive N to the environment. Current public investments to improve soil health seek to balance productivity and environmental considerations. However, data integrating soil biological health and crop N response to date is insufficient to reliably drive conservation policy and inform management. Here we used multilevel structural equation modeling and N fertilizer rate trials to show that biologically healthier soils produce greater corn yields per unit of fertilizer. We found the effect of soil biological health on corn yield was 18% the magnitude of N fertilization, Moreover, we found this effect was consistent for edaphic and climatic conditions representative of 52% of the rainfed acreage in the Corn Belt (as determined using technological extrapolation domains). While N fertilization also plays a role in building or maintaining soil biological health, soil biological health metrics offer limited *a priori* information on a site’s responsiveness to N fertilizer applications. Thus, increases in soil biological health can increase corn yields for a given unit of N fertilizer, but cannot completely replace mineral N fertilization in these systems. Our results illustrate the potential for gains in productivity through investment in soil biological health, independent of increases in mineral N fertilizer use.

## Introduction

Since the Green Revolution, nitrogenous mineral fertilizers have helped to nearly double global grain crop yields^1^. While this represents monumental gains in crop productivity, an estimated 41 to 50% of the N fertilizer applied to corn (*Zea mays* L.) globally since of the Green Revolution has been lost to the environment^2^, resulting in multifarious negative environmental effects^3–5^. Many strategies to address N losses from cropping systems are centered on the management of N fertilizer (e.g., “4Rs” of fertilizer management^6^), but neglect the role of soil^4,5^ biology in supplying plant N which often supplies over 50% plant N uptake in a growing season^7–9^. The framework of soil health seeks to highlight the critically important^10^, albeit inherently complex and uncertain^11,12^, role of soil biology in agroecosystems. Ultimately, soil health seeks to integrate soil biology with the historically emphasized chemical and physical soil components^13,14^. Soil health has been widely embraced by farmers, researchers, and private industry alike^15–18^. Additionally, soil health has also accrued broad legislative support in the form of nearly a dozen states incentivizing improved soil health as well as a Soil Health division within USDA^19^. Buy-in from these stakeholders represents a nexus of several key sources of information growers use in making nutrient management decisions^20–22^, as well as a demonstrated financial commitment.

While soil health has broad conceptual support, there is a dearth of empirical evidence connecting soil biological health measurements to vital soil functions or desired outcomes^23^. Although many studies have described management-induced changes in biological soil health indicators^24–28^, these indicators have only been loosely correlated with overall crop productivity^29–32^. Only recently have studies sought to integrate these metrics into nitrogen management strategies^33,34^. However, these studies used a single measurement of soil health and fertility and were conducted under a limited range of climatic and edaphic conditions. The clustering of data from similar sites can overestimate the strength of the relationships between the soil biological health indicators and crop productivity by confounding contextual effects with biological phenomena, ultimately increasing the potential for type I errors^35^. The use of multilevel models helps to reduce bias from data clustering, allowing us to differentiate between site-specific effects and a broader biological phenomenon. Therefore, while previous studies have established strong correlations, these correlations do not definitively link soil biological health and productivity because they do not account for potentially confounding factors^36^.

**Figure 1:**
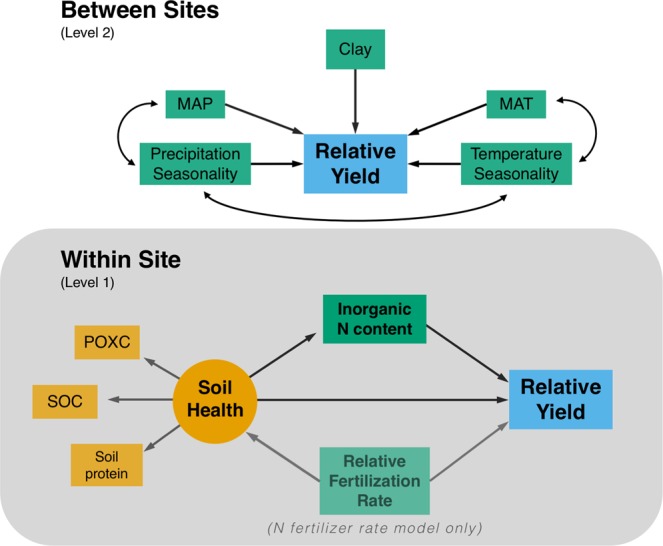
Baseline structural models for multilevel structural equation models. The N responsiveness model only included unfertilized check plots and therefore did not include the relative fertilization rate variable in that model. The N fertilizer rate model included all plots (including unfertilized plots). Both relative yield and relative fertilization rate were considered the yield and fertilization rate, respectively, relative to the calculated agronomic optimum. MAP = mean annual precipitation; MAT = mean annual temperature; POXC = permanganate oxidizable carbon; SOC = soil organic carbon.

In this study we use 29 replicated fertilizer N rate trials across the central and eastern Corn Belt of the Midwestern United States to evaluate the link between soil biological health and crop response to N fertilization by answering two essential questions. First, we answer the question “can soil biological health indicators predict the degree to which N fertilization is needed?” Secondly, we answer the broader question “do biologically active soils produce greater yields than less biologically active soils, for a given N fertilization rate?”. Due to the wide range of climatic and edaphic influences on relative yield with N fertilization, these primary research questions included site-level effects of climate and soil texture to better elucidate potential relationships between soil health and plant-soil N dynamics. Trials included in this study represented a variety of soil health-building managements across a range of edaphic and climatic conditions, allowing for inference across a breadth of contexts. To answer these questions, we constructed two multilevel structural equation models (Fig. 1)—referred to as the N responsiveness model and the N fertilizer rate model, respectively—to quantitatively define soil health and elucidate its relationships with crop-soil N dynamics (see Methods). We quantified overall crop productivity using relative yield—the ratio between the yield in a given experimental plot and the calculated agronomic optimum yield—where increasing relative yield indicates decreasing N responsiveness.

## Results & Discussion

### Soil health and responsiveness to N fertilization

**Figure 2:**
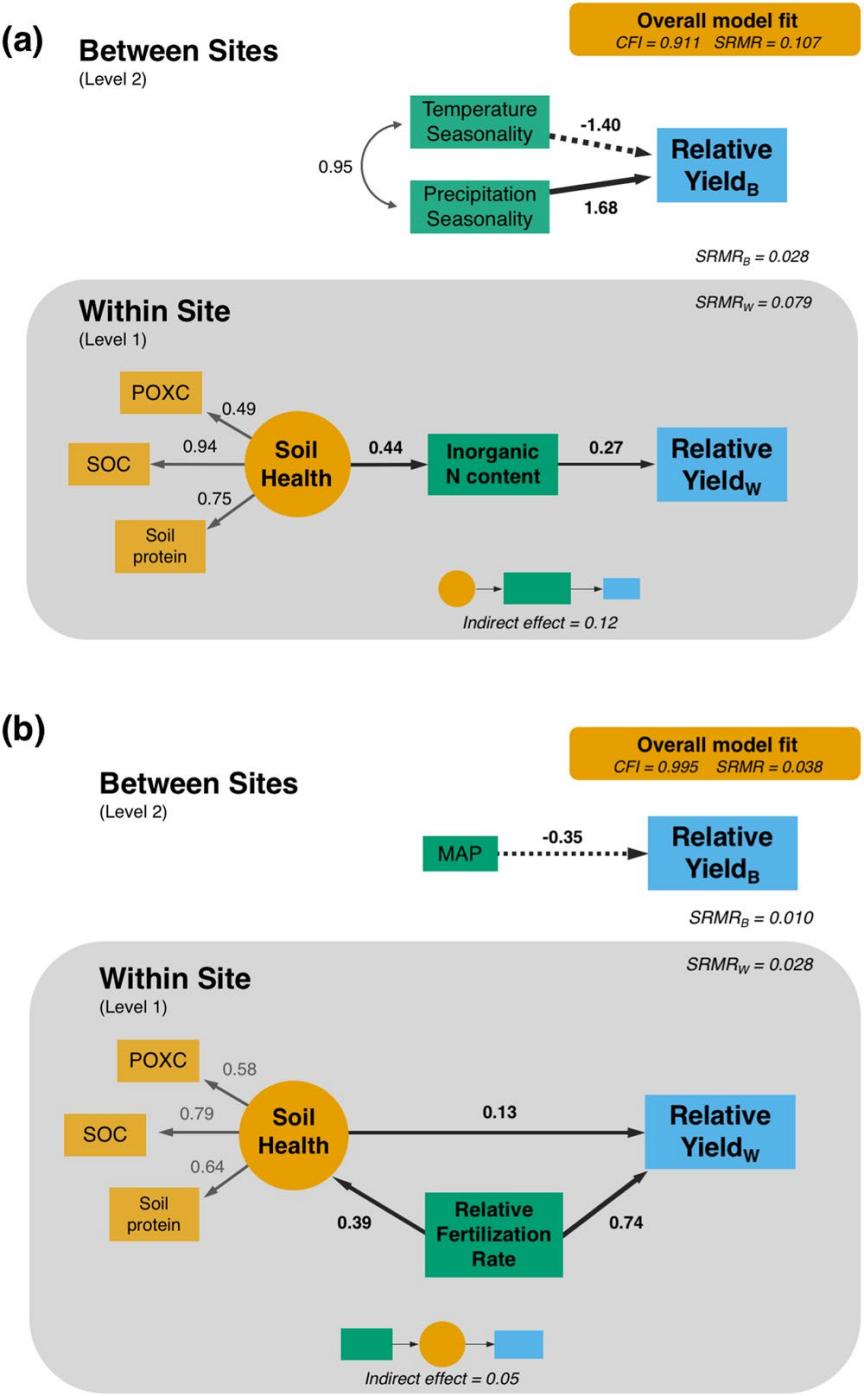
Finalized model for (**a**) N responsiveness model using only unfertilized check plots and (**b**) N fertilizer rate model using all plots. Relative yield_B_ and Relative yield_W_ are used to denote effects occurring on yields at the between sites and within-site levels, respectively. All path coefficients are standardized to represent effect sizes for significant relationships (p < 0.10). Model fit is assessed using the comparative fit index (CFI) and standardized root-mean-square of the residuals (SRMR), the latter of which is decomposed into error arising between sites (SRMR_B_) and within a site (SRMR_W_). Unstandardized regression coefficients and bootstrapped confidence intervals can be found in Tables S7 and S8, respectively.

The importance of pre-plant inorganic N in predicting crop response to N has resulted in its inclusion in many N recommendation frameworks^37^. Generally, preplant inorganic N is inversely related to crop N responsiveness (and positively related to relative yield), a result which is further validated in the current study. Here, inorganic N exerted a positive, direct effect on relative yield within any given site (β = 0.27, *p* = 0.049), underscoring its ubiquitous importance across a substantial portion of the Corn Belt (Fig. 2a). However, incorporating the positive direct effect of soil health on inorganic N content (β = 0.44, *p* < 0.0001) shows that soil biological health indirectly influences responsiveness to N (β = 0.12, *p* = 0.012). It is unexpected that soil biological health did not have a stronger, direct effect on relative yield. Previous work has shown that when inorganic N is well-supplied, biological soil health exerts an increasingly strong role due to other potentially limiting belowground resources that organic matter can supply^38,39^. However, the elevated error of model estimation (SRMR_W_ = 0.079) indicates considerable site-to-site variability in this relationship. Thus, adjustment for other soil characteristics and constraints, such as microbial community composition^40–42^ or mineralogy^11,43^ may be necessary to improve the accuracy of relationships between soil health and N responsiveness.

Although soil biological health and pre-plant inorganic N content influenced N responsiveness, the strongest influences were climatic variables (Fig. 2a). Between sites, there were considerable impacts of the seasonality of precipitation (β = 1.68, *p* = 0.021) and temperature (β = −1.40, *p* = 0.004) on relative yield in unfertilized plots. Bootstrapped 95% confidence intervals for all of these regression coefficients did not overlap with zero, indicating that our estimates were consistent in the direction of effect across the range of observations in our dataset. Thus, variability in precipitation among seasons in a year (i.e. seasonality) increased the relative yield in unfertilized check plots, whereas the seasonality of temperature decreased the relative yields. While our results are based on climatic averages, yearly variations in weather are also considerable sources of uncertainty in N management strategies. This is especially salient within the high rainfall regions of the Corn Belt^37^, and strategies to mitigate this uncertainty are imperative for decreasing agricultural N losses^3^. Nevertheless, the low model error across sites (SRMR_B_ = 0.028) suggests that these climatic factors strongly predict the degree to which a crop will be responsive to N fertilization.

### N fertilizer efficiency and soil biological health

Improvements in soil health increased relative yields per unit of fertilizer applied (Fig. 2b). The effect of soil biological health on relative yield (β = 0.13, *p* = 0.069) was 18% the magnitude of the fertilization effect (β = 0.74, *p* < 0.0001). Corn yields are driven by a suite of biotic and abiotic factors, but N availability is a primary driver of productivity. Given the critical role N fertilizer plays in maintaining yield, the finding that soil biological health accounts for nearly one fifth of the N fertilizer effect demonstrates the often-overlooked role that soil biological processes play in maintaining corn productivity in the Corn Belt. This increase in relative yield was consistent across the range of N fertilization, suggesting that the underlying processes are not simply a result of improved soil N supply to crops, but are instead indicative of other underlying processes as well. However, our results do not suggest what those processes might be. Nevertheless, our results imply that investments to improve soil biological health represents a considerable potential for improved environmental outcomes while maintaining farmer profitability^44^. The greater yields associated with improved soil biological health have been linked to lower residual N with concomitant decreases in absolute, as well as yield-scaled nitrate losses to surface water^45^ and N_2_O emissions^4,46^. Our findings are largely in agreement with a recent global meta-regression that showed both SOC and N fertilization rate having both independent and synergistic positive effects on crop yields^47^. In the current study, our low model error at the within-site level (SRMR_W_ = 0.029) demonstrates that this relationship is robust in a broad range of textural and climatic contexts. Additionally, our model demonstrates that biologically-based soil health indicators capture the beneficial effects of improved management on N fertility^48–51^. This validates the claims that: 1) these metrics are appropriate indicators of soil biological health, and 2) that a biologically healthy soil supplies a greater amount of nitrogen to crops.

Interestingly, higher N fertilization rates also increased soil biological health (β = 0.39, *p* < 0.0001; Fig. 2b). While there is a substantial debate in the literature regarding how or if mineral fertilizers influence soil C stocks and fluxes^52–55^, here we see evidence that N fertilization significantly improves soil biological health (Fig. 2b). While this may be due to increases in SOC (Table S6; r = 0.18, *p* = 0.002), N fertilization can also influence other more temporally-variable soil biological characteristics, such as microbial biomass^56^, plant residue composition^57^, and ligninolytic activity^58^. These parameters are not explicitly represented in our soil health factor, but they are indicative of alterations in C and N cycling that are highly related to our indicators of soil biological health^31,59–61^. The inferred alterations to C and N cycling are also evident in the differing relationship between soil biological health and relative yield in our models: soil biological health was not related to relative yield in the unfertilized plots of the N responsiveness model (Fig. 2a), but was evident in the fertilized plots of the N fertilizer rate model (Fig. 2b). This linkage between soil biological health and relative N fertilization rate implies that drastic decreases in N fertilization rates could have adverse effects on soil biological health and biological functioning. However, moderate decreases in N fertilization may be justifiable to optimize both yield and soil biological health^53^. Our results suggest that across N fertilization rates, improvements in soil biological health could lead to increases in productivity, while decreasing other externalities associated with losses of reactive N to the environment^62^. Further, these implications are broadly applicable, as they represent more than half of the rainfed corn acreage in the United States.

Between sites, the negative effect of MAP on relative yield (β = −0.35, *p* = 0.087) was unexpected in rainfed corn-based systems (Fig. 2b). Given the coarseness of this metric, this effect is likely attributable to an overall higher probability of N losses throughout the corn growing season^63^. This includes crucial periods where N supply exceeds crop demand, such as in the spring before crop establishment or in the fall after crop physiological maturity^64,65^. These N losses would decrease the amount of plant-available N—residual or from mineralized organic sources—ultimately decreasing relative yield at sub-optimal N fertilization rates.

### Lack of predictive ability of mineralizable C

Despite being the most common indicator of soil biological health^23^, mineralizable C (or flush of CO_2_ upon rewetting) was excluded from the soil health factor developed in our latent variable analysis. This represents a substantial deviation from a broad range of previous soil health literature relating mineralizable C to N mineralization^66,67^, crop response to N fertilization^33,34^, and to overall agronomic productivity^31,68^. To further justify this exclusion, we constructed permutations of our two final models wherein mineralizable C was included as an additional (exogenous) predictor of relative yield. These models were evaluated for model parsimony and absolute error (Table 1). For the N responsiveness model and the N fertilizer rate model, the overall RMSEA and SRMR_W_ values increased, with little to no decrease in SRMR_B_. Thus, the inclusion of mineralizable C resulted in considerable increases in the absolute error associated with both model fits, with most of the variation occurring at the site level. Similarly, AIC values increased with the inclusion of mineralizable C, indicating a much less parsimonious model. Taken together, this shows that mineralizable C made both models less accurate and less parsimonious, with the majority of that variation occurring on a site-by-site basis.

**Table 1:** Comparison of model fit indices with and without the inclusion of mineralizable C as an exogenous predictor variable of relative yield.

	N responsiveness		N fertilizer rate	
	With	Without	With	Without
CFI^a^	0.416	0.911	0.800	0.995
SRMR_W_^b^	0.197	0.079	0.148	0.028
SRMR_B_^c^	0.028	0.028	0.009	0.010
RMSEA^d^	0.308	0.162	0.219	0.052
AIC^e^	7635.1	6013.0	6363.4	3821.0

^a^CFI = comparative fit index; ^b^SRMR_W_ = standardized root mean square residual within site; ^c^SRMR_B_ = standardized root mean square residual between sites; ^d^RMSEA = root mean square error of approximation; ^e^AIC = Akaike Information Criteria.

Further evaluation of mineralizable C in a multi-site context was conducted by comparing the relationship between mineralizable C and relative yield in both a fixed and mixed-effects model. For both models, mineralizable C was considered a fixed effect, with the addition of “site” as a random variable in the mixed effects model. While mineralizable C has a statistically significant, positive relationship with relative yield in the fixed effect model, the mixed effects model showed that much of this was attributable to site-specific effects described by the random variable (Table 2). Aerobic respiration in surface soils is largely influenced by soil physiochemical characteristics, such as texture or bulk density^69–71^ and climatic variables^72^. Here, we see the strong relationship between clay content and mineralizable C as a likely contributor to this site-specific effect (Tables S5 and S6). These effects would operate at the between-sites level, rather than the within-site level, rendering the information provided by mineralizable C largely redundant in the context of a multilevel model.

**Table 2:** The relationships between mineralizable C and relative yield in a simple regression and a mixed effects model.

	Regression coefficient	F-value	Variance explained (R^2^)		Total
			Mineralizable C	Site (random)	
Simple regression	0.24	10.8^**^	0.031	—	0.031
Mixed effects model	0.07	0.6 ^ns^	0.002	0.362	0.364

Collectively, these results suggest that 1) mineralizable C provides less valuable information about relative yield than other soil biological health metrics and 2) that the information that mineralizable C does represent is largely attributable to inherent site characteristic. Therefore, mineralizable C is likely not a broadly applicable standalone predictor of relative yield across Corn Belt agroecosystems.

## Conclusions

This study was initiated to empirically test the broad-scale applicability of the soil health paradigm to N management in rainfed corn systems. Our results show that: 1) selected indicators of soil biological health can be used in conjunction with inorganic N content to predict the degree to which a site will be responsive to N fertilization, 2) improved soil biological health increases yields for a given fertilization rate, as soil health was 18% the magnitude of the fertilization effect on yields, and 3) that applications of N fertilizer can improve soil biological health. Additionally, our results suggest that the use of mineralizable C in N fertilization decisions is not robust across much of the US corn production acreage. Further, climatic influences exerted on a site-by-site basis are also highly influential for soil-plant N dynamics. Our use of multi-level modelling allows for inference beyond our specific sites to conditions that are representative of approximately half of US rainfed corn systems.

## Methods

### Study details

Soil and yield data from replicated N rate studies located across the Corn Belt (n = 29 sites; n = 386 total soil samples) were collected from 2015–2018. Trials had a minimum of four imposed N rates, each of which was replicated 3 to 6 times. Each replication had an unfertilized control plot without applied N as well as a minimum of three additional N rates, the highest of which was at least 180 kg ha^−1^ N. The one exception was the Purdue N Trials, in which the check plots had an N rate of 24–28 kg ha^−1^ N applied at planting. Layered within these N rate trials were additional soil health-building management strategies: varying rotation lengths and diversities, manure application, cover crops, and decreases in tillage intensity (Table S1). Yield data from these trials was limited to years in which growing season precipitation was not a limiting factor (i.e. non-drought years).

These studies represented both on-farm and research plots covering a broad range of climatic and edaphic conditions (Table S2). Using GPS coordinates for each site, we extracted 30 year average climatic data for each site from the WorldClim2 database^73^ using RStudio^74^ and the *raster* package^75^. We then used this data to describe climatic influences on relative yield across sites. We determined the representativeness of our selected sites using technological extrapolation domains (TEDs)^76,77^, which are constructed using a combination of climatic and edaphic factors to determine yield potential for rainfed cropping systems. With the TED framework, our sites represented 52% of the total rainfed corn production area in the central/eastern United States.

Soil samples for each study were gathered in the spring prior to or immediately following planting of the corn grain crop, but prior to fertilizer application. We took multiple soil subsamples to a depth ranging between 15 and 30 cm, depending on the study, which were then composited and homogenized to comprise one representative sample for each plot. Samples were then air-dried and stored pending analysis. The specific sampling protocols for each study can be found within references cited in the Supplementary Information (Tables S1 and S9).

### Soil nutrient status

We determined soil inorganic N content as the sum of both nitrate and ammonium. Both were determined using a 1:5 soil:solution extract using 1 or 2 M KCl. Extracts were shaken for 1 hour, clarified, and measured colorimetrically^78^. Ammonium was determined using the salicylate method^79^. Nitrate was determined using the cadmium reduction method^80,81^ or by reduction with vanadium (III) chloride^82^.

### Soil health metrics

As an aggregated measure of soil biological health, we selected three commonly-utilized metrics recommended by the USDA^83^ and Cornell’s Comprehensive Assessment of Soil Health^84^. Permanganate oxidizable carbon (POXC)^24,85^, autoclave citrate-extractable protein (which we refer to hereafter as “soil protein”)^86^, and mineralizable C^87,88^ were determined for each sample. These metrics were selected using two criteria: 1) the ability of the method to be adapted to the high-throughput context of commercial agronomy labs and 2) being conceptually representative of a component of biological N cycling processes. In accordance with the USDA recommendations for soil health metrics^83^, we considered mineralizable C to be an indicator of general microbial activity, POXC to represent a readily available C pool, and soil protein to represent a bioavailable N pool. In addition to the biological soil health indicators, we also measured organic matter content, as soil organic matter is the most commonly measured metric associated with soil health^23^ and is representative of overall C cycling^83^. Total organic matter content was measured using either loss-on-ignition (LOI)^89^ or total C on combustion^90^. Wherever both combustion and LOI values were available, total C values were used^91^. Where only LOI values were available, LOI was converted to total C, or soil organic carbon (SOC), using the conversion factor of 1.74^92^ to facilitate comparisons with the broader literature. Detailed description of laboratory analyses can be found in the SI Methods.

### Statistical analyses

Our overall statistical approach consisted of several steps. First, we used a factor analysis to determine the appropriate number of “soil health” factors, as well as which measured variables were to be considered. Next, this soil health factor was integrated into a theoretical model describing the relationships between soil biological health, soil inorganic N content, and relative yield. This theoretical model (represented in Level 1 of our model; Fig. 1) was then fit into a multilevel context, wherein site-specific effects (i.e. texture and climate) were used to more accurately constrain and quantify these relationships at the between sites level (Level 2).

We used exploratory factor analysis (EFA), or common factor analysis, to determine if the soil biological health indicators describe similar underlying processes. The procedure of EFA uses the pattern of correlations (i.e. covariance structure) of a set of measured variables to infer underlying processes or constructs^93,94^. The goal of EFA is distinctly different from other data reduction processes (e.g. principal components analysis) in that EFA is attempting to describe unmeasurable latent constructs. The classic example of this is the concept of “general intelligence” being represented by a combination of measured test scores^95^. Here, EFA was performed using the selected indicators of soil biological health and fertility—POXC, mineralizable C, soil protein, and soil organic C content—to determine if these measured variables describe similar latent constructs. To determine the appropriate number of factors, we used the package *nFactors*^96^ in RStudio^74^ to conduct several quantitative assessments for factor retention, all of which suggested a best fit of one factor to describe soil health. This suggests that these indicators are all describing some portion of the underlying construct of “soil health”. However, each indicator differed in its ability to describe this underlying soil health construct; a factor loading < 0.50 and correlation with other measured variables < 0.50 (Tables S5 and S6) led us to exclude mineralizable C from the final latent factor (Table S4).

We used multilevel structural equation models to determine the effects of soil biological health on crop N responses. Multilevel models—also referred to as hierarchical models—offer many advantages over classical regression models in broad-scale studies such as the current study^97^: simultaneous quantification of effects across scales, the balancing of type I and type II errors, and accuracy of model prediction to similar sites (see Supplementary Information for further discussion). Here, we use multilevel models to differentiate between effects that are exerted at the site level (i.e. between sites or level 2 of our analysis) and effects that are exerted within a site (i.e. within site of level 1 of our analysis). To answer the two primary questions surrounding crop response to N fertilization, we built two similar, but separate models (Fig. 1). Our first model was constructed to address the question “can biological soil health indicators predict the degree to which N fertilization is needed?”. This model, referred to hereafter as the “N responsiveness model”, was conducted using only soil measurements from our unfertilized check plots. Associated yields from those check plots were then used as the numerator and yields at the agronomic optimum N rate (AONR) as the denominator in calculating relative yield. We constructed our second model to answer the question “do biologically active soils produce greater relative yields than less biologically active soils, for a given N fertilization rate?”. This model, which we will refer to as the “N fertilizer rate model”, included all soil and yield data available. We analyzed our models using the *sem()* command in the *lavaan* package^98^ of RStudio with our data clustered at the site level. Using our constructed baseline model, paths were iteratively eliminated based on their level of significance until each individual path was significant at p < 0.10. Overall model fit was assessed using the standardized root-mean-square of the residuals (SRMR) as a measure of absolute fit and the Comparative Fit Index (CFI) and Akaike’s Information Criteria (AIC) as measures of relative fit^99,100^. Bootstrapped confidence intervals were used in model validation^101^ and to assess indirect effects^102^. Details of model evaluation and justification can be found in the Supplementary Information (SI Methods).

## Supplementary information

Supplementary Information and Methods.

## Data Availability

Data is currently being used for another work and is available upon request from the corresponding author.
