# Supplementary material for: Improved soil biological health increases corn grain yield in N fertilized systems across the Corn Belt
Source: Sci Rep. 2020 Mar 3;10:3917. doi: 10.1038/s41598-020-60987-3 (PMC7054259; doi:10.1038/s41598-020-60987-3)
Supplement: Supplementary file 1 — Supplementary Information and Methods. [file 41598_2020_60987_MOESM1_ESM.docx]

**Supplementary Information for**

**Title:** Improved soil biological health increases corn grain yield in N fertilized systems across the Corn Belt

**Authors:**

Jordon Wade ^1,2^*, Steve W. Culman ^1^, Jessica A.R. Logan ^3^, Hanna Poffenbarger ^4^, M. Scott Demyan ^1^, John H. Grove ^4^, Antonio P. Mallarino ^5^, Joshua M. McGrath ^4^, Matthew Ruark ^6^, and Jaimie R. West ^6^

^1^ School of Environment & Natural Resources, The Ohio State University; ^2^ Department of Crop Sciences, University of Illinois, Urbana-Champaign; ^3^ College of Education and Human Ecology, The Ohio State University; ^4^ Department of Plant and Soil Sciences, University of Kentucky; ^5^ Department of Agronomy, Iowa State University; ^6^ Department of Soil Science, University of Wisconsin — Madison

*corresponding author: [jordonwade@gmail.com](mailto:jordonwade@gmail.com)

**Supplementary Information Text**

**Methods**

Soil analyses

We measured mineralizable C—also referred to as respiration, the flush of CO_2_ upon rewetting or potentially mineralizable C—by rewetting 10g of air-dried soil in 50 mL microcosms to 50% water-filled pore space, incubating for 24 hours at a constant temperature of 25°C, and measuring CO_2_ concentrations on a 1mL aliquot of headspace on a LI-COR LI-820 infrared gas analyzer (LI-COR Biosciences, Lincoln, NE). We calculated mineralizable C as the difference in CO_2_ concentration between a sample and a blank control using the ideal gas law ^1,2^. In the statistical models, the mean value of duplicate measurements was used to account for potential measurement variability ^3^. We measured POXC using 2.5 g of air-dried soil, which was shaken for precisely 2 minutes in a 0.02 mol L^-1^ KMnO_4_ solution, allowed to settle for 10 minutes, diluted 1:100 with water, and then measured spectrophotometrically at 550 nm on a 96-well plate. We measured autoclave-citrate extractable protein—which we refer to as “soil protein”—using 3.0 g of air-dried soil that was extracted with 0.02 mol L^-1^ sodium citrate solution (pH=7.0), shaken for 5 minutes, and autoclaved at 121°C (15 psi) for 30 minutes. Cooled extracts were then clarified (centrifuged @ 10,000 × g for 3 minutes) and reheated (60°C for 1 hour) after adding a bicinchoninic acid reagent. After a second cooling for 5 minutes, we quantified soil protein colorimetrically at 562 nm using a bovine serum albumin standard ^4^. Organic matter content was measured using the recommended method for the region: loss-on-ignition (LOI) ^5,6^. In brief, soil was dried overnight and then combusted at 360°C for 2 hours, with the difference being representative of total organic matter content.

Statistical methods and model development

In the process of exploratory factor analysis (EFA), the selection of the number of factors to retain from a dataset requires careful consideration ^7,8^. EFA draws heavily upon eigenvalues, a measure of the amount of “information” (i.e., variance of observed variables explained) within a factor. Therefore, an eigenvalue > 1.0 contains more information than an observed variable, providing justification for using factor analysis to decrease dimensionality. Early methods of factor selection involved the visual inspection and the retention of factors prior to a “substantial drop” in eigenvalues ^9^, introducing subjective definitions of which drops were or weren’t “substantial”. Subsequent quantitative methods of evaluating a “substantial drop” in eigenvalues have been developed to guide factor retention ^10^. However, factor selection is still a delicate process, especially when quantitative selection methods do not converge ^7^. Here, we used four quantitative, eigenvalue-based factor selection methods from the *nFactors* package ^11^: the Kaiser criterion, optimal coordinates, acceleration factor, and parallel analysis. In our selection of the number of “soil health” factors to retain, all four methods suggested that retaining one factor was the most parsimonious fit of our data (Figure S2).

Next, we performed a factor analysis using the *fa( )* command in the *psych* package ^12^ to determine the loadings of the observed variables onto the one factor. Using maximum likelihood and a varimax rotation, standardized loadings of observed variables ranged from 0.48 to 0.71 (Table S4). While there is no set threshold of acceptable values for retained versus excluded values, 0.40 is considered a more liberal threshold and 0.60 a more conservative threshold ^13^. We used a threshold of 0.50 to reflect a balance of these two considerations, leading us to exclude mineralizable C from the factor in the final structural model. This decision was further strengthened by the low correlations of mineralizable C with other biological soil health indicators (Tables S5 and S6), with the exception of the relationship with POXC in the unfertilized plots of the N responsiveness model (Table S5).

To determine the effect of soil health across sites, we integrated the soil health factor into a multilevel linear regression model, which offers numerous advantages over other regression-based techniques. First, the multilevel structure accounts for varying effects to be simultaneously quantified across several scales of analysis. The hierarchically lower level in our current model was the “within site” portion of the model and the hierarchically higher level was the “between sites” (or “across sites”) portion of the model. Within-site models are comprised of components that inform N management decisions (e.g. inorganic N content). In order to accommodate for variations in yield potential for each site, we allowed the intercept to vary (as a random effect), but included measured model predictors as fixed effects. The upper level of our model—the between- or across-sites effects—were parameters that would exert their influence on a site-by-site basis (e.g. climate conditions). Thus, we could estimate within-site changes in N dynamics while holding climatic or edaphic parameters constant between-sites. This alludes to a second advantage of multilevel modeling over classical regression: prediction to new groups drawn from the same sampling population. In accounting for variations between/across sites, prediction in new sites is more robust and reliable. A third salient advantage of multilevel models is the balancing of type I and type II errors in multi-site studies. Multi-site studies will often include all observations in a single predictive regression from which to draw conclusions, often referred to as “complete pooling”. While this results in a larger sample size, clustering within sites tends to overestimate the strength of that relationship, increasing the potential for Type I errors (false positives). Alternatively, many multi-site studies will average parameters across replicates within a site and then use that average in regression-based evaluations. This approach decreases the overall sample size, resulting in an underpowered analysis and an increase in the probability of a Type II error. The partial pooling method of multilevel analysis balances these two considerations, producing a more accurate error estimate while maintaining an appropriate balance of type I versus type II errors.

Relative yield—the between the yield in a given plot and the yield at the agronomic optimum N fertilization rate (AONR)—was selected as the response variable of interest in each mode. For the N responsiveness model, relative yield was calculated using only unfertilized yields in check plots, where a higher relative yield indicates less responsiveness to N fertilization For the model to determine the effect of N fertilization rate, relative yield was calculated as the ratio of yield at that plot’s N rate and the yield at AONR. The AONR was calculated using the linear, linear plateau, quadratic, and quadratic plateau methods of Cerrato and Blackmer ^14^. Model fit (R2) was used to determine the appropriate response curve, with an increase of >0.05 being needed to justify any variations from the preferred quadratic plateau model. Yield was then calculated using the AONR and resulting yield response curve. If calculated AONR exceeded the highest fertilization rate, then yield at the highest N fertilization rate was used. AONR was calculated independently for each treatment by block combination to account for: 1) potential long-term effects of treatment on AONR ^15^ and 2) within-site spatial variation in yield potential ^16^. The relative yield was then determined for each treatment by block combination and expressed as a percentage, where non-responsive sites have a value of relative yield =100%. In the long-term studies (N Rotation, Blevins, and F05), yields were averaged across years (up to 5 years) to minimize year-to-year weather effects on N response. In the single-year studies, grain yields were used from the harvest following spring soil sampling. To avoid potentially confounding effects of low rainfall years, we only used yield data from years that was considered representative of a given site. Due to high variability in optimum N rate between sites ^17^, a relative fertilization rate was used to compare N rate across sites. Relative fertilization rate was calculated as the applied N rate divided by the AONR. Thus, some plots had relative fertilization rates > 1.0, indicating excess N fertilization.

Overall model fit was assessed using the standardized root-mean-square of the residuals (SRMR) as a measure of absolute fit and the Comparative Fit Index (CFI) and Akaike’s Information Criteria (AIC) as measures of relative fit ^18,19^. Combining CFI and SRMR, we used the cutoff criteria of Hu and Bentler ^18^, wherein either a CFI > 0.95 or a SRMR < 0.05 was considered a close model fit. For CFI < 0.95 and SRMR > 0.05, we would expect a combined type I and type II error rate of ~1% for n ≈ 200 and ≤ 0.0% for n ≈ 400. We determined fit separately at each level using SRMR ^20^ with a similar criteria of SRMR < 0.05 being considered a close fit and SRMR < 0.08 being an acceptable fit. Values of SRMR > 0.10 are considered to be a mediocre model fit ^21^. To ensure the robustness of our results, we used bias-corrected and accelerated (BCa) bootstrapping to construct parameter confidence intervals during model development and validation ^22^, as well as to assess indirect effect (i.e. mediation) ^23^.

Theoretical justification of statistical model

When using structural equation models, a robust theoretical underpinning is necessary to testing the causal hypotheses of the underlying structural model ^24^. The following constitutes the theoretical bases of our models. The N responsiveness model and the N fertilizer rate model shared similar general model structures. At the lower (within-site) level of our model we included the long-established relationship between pre-plant inorganic N content and relative corn yield ^25,26^. One of the primary relationships of interest—the relationship between the soil health factor comprised of biological soil health indicators—was also hypothesized to have a direct effect on relative yield. These structural components—the soil health factor, inorganic N, and relative yield—were identical across models. However, the integration of fertilizer rate allowed for the interactive effects of fertilizer application and soil health on relative yield to be determined. Given the influence of nitrogenous fertilizer application on soil microbial biomass and activity ^27,28^ and its effects on soil carbon dynamics ^15^, we hypothesized that our soil health factor would also be influenced by N fertilization rates. At the upper level of our model, we included both textural (e.g. clay content) and climatic variables that been shown to influence crop N response ^29,30^.

**Results**

Soil biological health and physiochemical properties

Soil physiochemical properties represented a wide range of characteristics (Table S2). Clay contents represented by these soils varied by as much as an order of magnitude, ranging from 43 to 436 g kg^-1^. CEC values ranged from 2.0 to 25.9, encapsulating a wide range of soil fertility status. Similarly, pH for each study were circumneutral to acidic. However, in this range, pH values are not expected to substantially influence yield ^31^. Notably, nearly all of the SOC values fell below the ~2% SOC threshold that Oldfield et al. ^31^ estimated as a plateau for yield increases with N fertilization.

There are few studies with regionally-representative values for biological soil health indicators, so determining “high” vs. “low” values is often difficult. However, the samples in the current study exhibited a generally wide range of values (Table S3). The POXC values ranged from 160 to 1054 mg kg^-1^ soil; around one order of magnitude. Similarly, mineralizable C ranged from 5.8 mg CO_2_-C kg^-1^ soil to 167.1 mg CO_2_-C kg^-1^ soil. This represents a broader range of mineralizable C values than was recorded by Franzluebbers et al. ^32^, despite the narrower range of climatic conditions in the current study. Soil protein values ranged from 1.7 to 7.1 g kg^-1^ soil, which was generally within expected values for medium or fine-textured soils ^33^.

Relationships between biological soil health, agronomic, and climatic variables

In both the reduced dataset (N responsiveness) and the full dataset (N fertilizer rate), many of the soil health and agronomic variables were significantly linearly correlated to one another (Table S5 and S6). Interestingly, mineralizable C was largely unrelated to many of the climatic variables and only weakly related to other soil health indicators in both datasets. However, it was moderately related to clay content (r ≈ 0.50). Climatic variables were largely well-correlated with one another, with many of the relationships r > 0.90. Both MAT and MAP were inversely related to their seasonality components. Thus, our warmer sites had less variability in their temperature between seasons and our wetter sites demonstrated more consistent monthly precipitation. Across datasets, very few of the soil health or climatic variables exhibited strong relationships with relative yield, except relative fertilizer rate in the full dataset. Collectively, these interrelationships represent substantial multi-collinearity within both the reduced and full datasets.


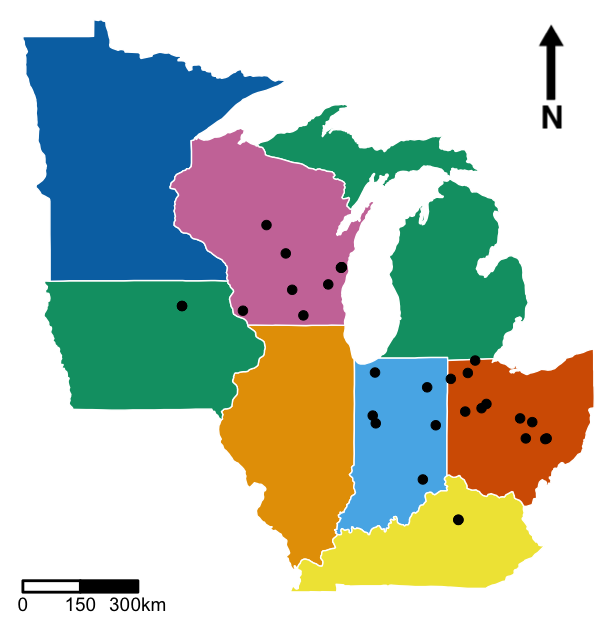


**Fig. S1.** Map of all sites included in the current study.


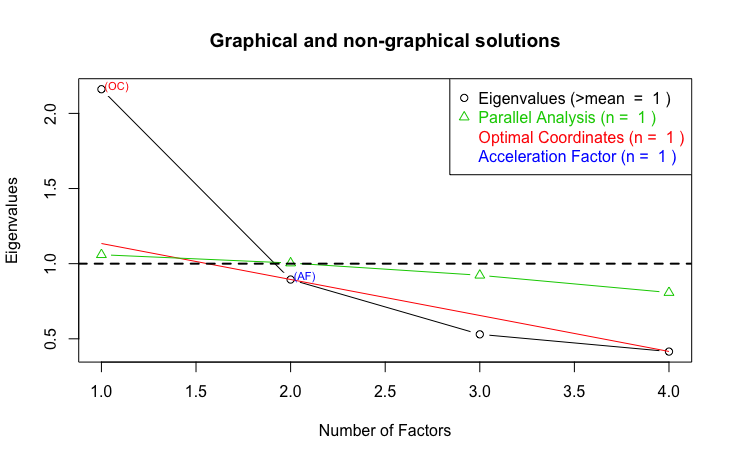


**Fig. S2.** Scree plot to select the number of factors to retain from the exploratory factor analysis. The dotted line indicates the eigenvalue = 1.0 threshold used in Kaiser’s criterion.

**Table S1.** Location and primary management information for all study sites. All sites had a minimum of four N fertilization rates. Additional management information can be found in Table S9 or in references cited in table.

| **Study Name** | **Treatment(s)** | **State** | **Number of Sites** | **Number of total plots** | **Treatment Duration** | **References** |  |
| --- | --- | --- | --- | --- | --- | --- | --- |
| N Rotation | Four rotations: continuous corn (grain), corn-soybean, and corn-corn-corn-soybean | Iowa | 1 | 66 | 36 years | ^34^ |  |
| Manure × CC^a^ | Non-manured control, manure only, manure + barley CC | Wisconsin | 4 | 48 | 2 years | None |  |
| Radish CC | No CC control, radish CC, radish CC + pre-plant N^b^ | Wisconsin | 3 | 28 | 3 years | ^35^ |  |
| Purdue N Trials | None | Indiana | 6 | 90 | 4 years | ^36^ |  |
| Blevins | Moldboard plow and continuous no-till | Kentucky | 1 | 32 | 48 years | ^37^ |  |
| F05 | Continuous corn (grain), corn-soybean rotations | Kentucky | 1 | 48 | 35 years | None |  |
| Legume CC | No CC control, clover CC, barley CC | Wisconsin | 3 | 36 | 1 year | None |  |
| Ohio N Trials | On-farm trials (varies) | Ohio | 12 | 36 | 1 year | None |  |
| ^a^ CC= cover crop; ^b^ 67 kg N ha^-1^ was added at during radish planting | | | | | | | |

**Table S2.** Climatic and edaphic data for each site.

| Study Name | Site Number | Clay (g kg^-1^ soil) | MAT^b^ (°C) | MAP^c^ (mm) | Temperature seasonality^d^ (°C) | Precipitation seasonality^e^ (%) |
| --- | --- | --- | --- | --- | --- | --- |
| N Rotation | 1 | 225 | 7.6 | 858 | 11.2 | 4.8 |
| Manure × CC^a^ | 2 | 229 | 7.4 | 806 | 10.6 | 3.9 |
|  | 3 | 128 | 5.5 | 837 | 11.0 | 4.5 |
|  | 4 | 43 | 6.4 | 809 | 10.8 | 4.2 |
|  | 5 | 227 | 7.9 | 846 | 10.6 | 4.2 |
| Radish CC^a^ | 6 | 147 | 7.1 | 812 | 10.4 | 3.7 |
|  | 7 | 247 | 7.3 | 833 | 10.2 | 3.2 |
|  | 8 | 225 | 8.0 | 850 | 10.5 | 3.6 |
| Purdue N Trials | 9 | 369 | 10.1 | 943 | 9.7 | 2.5 |
|  | 10 | 436 | 9.8 | 944 | 9.5 | 2.2 |
|  | 11 | 296 | 11.9 | 1102 | 8.9 | 1.7 |
|  | 12 | 323 | 9.5 | 973 | 9.7 | 2.5 |
|  | 13 | 280 | 10.3 | 962 | 9.6 | 2.3 |
|  | 14 | 273 | 9.5 | 947 | 9.6 | 2.2 |
| Blevins | 15 | 180 | 12.5 | 1148 | 8.5 | 1.5 |
| F05 | 16 | 179 | 12.5 | 1140 | 8.6 | 1.5 |
| Legume CC^a^ | 17 | 229 | 7.4 | 824 | 10.1 | 3.2 |
|  | 18 | 221 | 7.4 | 824 | 10.1 | 3.2 |
|  | 19 | 223 | 7.3 | 833 | 10.2 | 3.2 |
| Ohio N Trials | 20 | 240 | 10.0 | 899 | 9.6 | 2.1 |
|  | 21 | 140 | 10.6 | 974 | 8.7 | 2.0 |
|  | 22 | 141 | 10.5 | 978 | 8.8 | 2.0 |
|  | 23 | 286 | 9.9 | 909 | 9.5 | 2.2 |
|  | 24 | 191 | 9.5 | 1016 | 9.0 | 2.0 |
|  | 25 | 178 | 9.2 | 1014 | 9.1 | 2.1 |
|  | 26 | 288 | 9.3 | 867 | 9.6 | 2.1 |
|  | 27 | 195 | 10.4 | 1012 | 9.0 | 2.0 |
|  | 28 | 253 | 9.2 | 853 | 9.5 | 2.0 |
|  | 29 | 389 | 9.4 | 872 | 9.6 | 2.0 |
| ^a^ cover crop; ^b^ mean annual temperature; ^c^ mean annual precipitation; ^d^ calculated as the standard deviation of the average monthly temperature; ^e^ coefficient of variation for monthly total precipitation within a year | | | | | | |

**Table S3.** Mean values for soil fertility, soil health, and physiochemical characteristics in each study. Values in parentheses indicate the minimum and maximum values within each study, respectively.

| **Study Name** | **pH** | **SOC (%)** | **CEC**  **(meq/100g soil)** | **Inorganic N (mg N/kg soil)** | **POXC**  **(mg/kg soil)** | **Mineralizable C (mg CO_2_-C/kg soil)** | **Soil Protein**  **(g/kg soil)** |
| --- | --- | --- | --- | --- | --- | --- | --- |
| N Rotation | 5.7 (4.6, 6.7) | 1.5  (0.8, 2.2) | 14.2 (10.0, 18.8) | 55.2  (8.7, 149.2) | 533  (431, 623) | 48.7  (26.2, 79.1) | 3.8  (2.8, 5.4) |
| Manure × CC^a^ | 6.6 (5.9, 7.2) | 1.2  (0.1, 2.1) | 9.8  (2.0, 17.8) | 11.0  (4.6, 23.4) | 450  (160, 810) | 35.2 (5.9, 92.7) | 3.3 (1.7, 7.1) |
| Radish CC | 7.0 (6.4, 7.5) | 1.4  (1.1, 1.8) | 12.4  (9.8, 16.1) | 7.6 (4.3, 18.1) | 518 (347, 710) | 47.3 (27.0, 69.0) | 3.0 (2.3, 4.2) |
| Purdue N Trials | 6.2 (5.4, 7.3) | 1.9  (1.1, 3.1) | 14.6  (7.3, 25.9) | 14.2  (3.1, 32.2) | 570 (291, 821) | 59.4  (22.8, 167.1) | 3.9  (2.9, 5.9) |
| Blevins | 5.5 (4.9, 6.3) | 1.5  (1.0, 2.1) | 11.6  (10.3, 13.4) | 6.1  (3.6, 14.7) | 620  (398, 921) | 40.8  (13.4, 75.5) | 4.7 (3.5, 6.5) |
| F05 | 5.0 (4.0, 5.8) | 1.5  (1.3, 1.9) | 12.7 (10.1, 16.7) | 8.4  (4.9, 32.5) | 565  (347, 1054) | 45.0  (11.3, 73.5) | 4.2  (3.3, 5.8) |
| Legume CC | 7.4 (6.8, 7.6) | 1.1  (0.8, 1.9) | 13.2 (11.1, 16.7) | 7.4 (3.7, 13.3) | 527 (370, 761) | 50.0 (22.4, 81.8) | 2.7 (1.8, 4.2) |
| Ohio N Trials | 6.1 (4.8, 6.9) | 2.2  (1.6, 6.4) | 11.7 (5.3, 19.3) | 21.8  (9.7, 47.9) | 473  (309, 761) | 33.0  (5.8, 55.4) | 4.6  (3.5, 6.3) |
| ^a^ CC = cover crop | | | | | | | |

**Table S4.** Estimated loadings of observed variables onto latent soil health factor.

| **Observed Variable** | **Factor Loading** | **Communality (h^2^)** | **Uniqueness (u^2^)** |
| --- | --- | --- | --- |
| Mineralizable C | 0.48 | 0.23 | 0.77 |
| Soil protein | 0.61 | 0.37 | 0.63 |
| Soil organic C | 0.70 | 0.49 | 0.51 |
| POXC | 0.71 | 0.50 | 0.50 |

**Table S5.** Correlation matrix for all measured variables in the N responsiveness model (n=186).

|  | **Inorg N** | **Min C** | **POXC** | **Soil protein** | **SOC** | **Clay** | **MAT** | **TS** | **MAP** | **PS**^i^ |  |
| --- | --- | --- | --- | --- | --- | --- | --- | --- | --- | --- | --- |
| **RY**^a^ | 0.23^**^ | *0.10* | 0.12^†^ | 0.16^*^ | *0.07* | 0.13^†^ | -0.17^*^ | 0.19^**^ | -0.18^*^ | 0.15^*^ |  |
| **Inorg N**^b^ |  | *-0.06* | *0.02* | 0.39^***^ | 0.36^***^ | *0.08* | 0.13^†^ | *-0.05* | 0.15^*^ | *-0.03* |  |
| **Min C**^c^ |  |  | 0.67^***^ | 0.24^***^ | 0.25^***^ | 0.45^***^ | *-0.04* | *0.10* | *-0.01* | *0.07* |  |
| **POXC**^d^ |  |  |  | 0.44^***^ | 0.46^***^ | 0.39^***^ | *0.05* | *-0.04* | *0.06* | *-0.08* |  |
| **Soil protein** |  |  |  |  | 0.66^***^ | 0.20^**^ | 0.34^***^ | -0.33^***^ | 0.38^***^ | -0.34^***^ |  |
| **SOC**^e^ |  |  |  |  |  | 0.60^***^ | 0.40^***^ | -0.34^***^ | 0.31^***^ | -0.40^***^ |  |
| **Clay** |  |  |  |  |  |  | 0.34^***^ | -0.21^**^ | 0.12^†^ | -0.32^***^ |  |
| **MAT**^f^ |  |  |  |  |  |  |  | -0.87^***^ | 0.92^***^ | -0.83^***^ |  |
| **TS**^g^ |  |  |  |  |  |  |  |  | -0.82^***^ | 0.98^***^ |  |
| **MAP**^h^ |  |  |  |  |  |  |  |  |  | -0.74^***^ |  |
| †, *, **, and *** correspond to p-values of <0.10, <0.05, <0.01, and <0.001, respectively. Italicized values are not significant at p<0.10.  ^a^ relative yield; ^b^ inorganic N; ^c^ mineralizable C; ^d^ permanganate oxidizable C; ^e^ soil organic carbon; ^f^ mean annual temperature; ^g^ temperature seasonality; ^h^ mean annual precipitation; ^I^ precipitation seasonality | | | | | | | | | | | |

**Table S6.** Correlation matrix for all measured variables in the N fertilizer rate model (n=384).

|  | **RFR** | **Inorg N** | **Min C** | **POXC** | **Soil protein** | **SOC** | **Clay** | **MAT** | **TS** | **MAP** | **PS^j^** |
| --- | --- | --- | --- | --- | --- | --- | --- | --- | --- | --- | --- |
| **RY**^a^ | 0.69^***^ | 0.19^***^ | 0.18^*^ | 0.25^***^ | 0.32^***^ | 0.18^***^ | 0.21^***^ | 0.23^***^ | -0.13^*^ | 0.23^***^ | -0.15^**^ |
| **RFR**^b^ |  | 0.20^***^ | 0.12^*^ | 0.29^***^ | 0.48^***^ | 0.18^**^ | 0.15^**^ | 0.49^***^ | -0.35^***^ | 0.49^***^ | -0.35^***^ |
| **Inorg N**^c^ |  |  | -0.12^*^ | -0.03 | 0.20^***^ | *0.07* | *0.02* | -0.24^***^ | 0.42^***^ | -0.22^***^ | 0.45^***^ |
| **Min C**^d^ |  |  |  | 0.45^***^ | 0.13^*^ | 0.33^***^ | 0.51^***^ | *0.02* | *0.01* | *0.00* | *-0.03* |
| **POXC**^e^ |  |  |  |  | 0.43^***^ | 0.45^***^ | 0.22^***^ | 0.18^***^ | -0.15^**^ | 0.19^**^ | -0.17^***^ |
| **Soil protein** |  |  |  |  |  | 0.50^***^ | *0.06* | 0.46^***^ | -0.39^***^ | 0.49^***^ | -0.37^***^ |
| **SOC**^f^ |  |  |  |  |  |  | 0.59^***^ | 0.28^***^ | -0.22^***^ | 0.19^***^ | -0.28^***^ |
| **Clay** |  |  |  |  |  |  |  | 0.12^*^ | *-0.05* | *-0.04* | -0.17^**^ |
| **MAT**^g^ |  |  |  |  |  |  |  |  | -0.92^***^ | 0.96^***^ | -0.88^***^ |
| **TS**^h^ |  |  |  |  |  |  |  |  |  | -0.89^***^ | 0.98^***^ |
| **MAP**^i^ |  |  |  |  |  |  |  |  |  |  | -0.82^***^ |
| †, *, **, and *** correspond to p-values of <0.10, <0.05, <0.01, and <0.001, respectively. Italicized values are not significant at p<0.10.  ^a^ relative yield; ^b^ relative fertilization rate; ^c^ inorganic N; ^d^ mineralizable C; ^e^ permanganate oxidizable C; ^f^ soil organic carbon; ^g^ mean annual temperature; ^h^ temperature seasonality; ^I^ mean annual precipitation; ^j^ precipitation seasonality | | | | | | | | | | | |

**Table S7.** Regression coefficients and bootstrapped confidence interval of each relationship in the final N responsiveness model (n_B_ = 29 sites; n_W_ = 186 samples^a^). Italicized values indicate factor loadings rather than regression coefficients.

| **Level** | | **Regression** | **Standardized**  **estimate (β)** | **Unstandardized**  **estimate (B)** | **95% CI**  **(unstandardized)** | **p-value** |
| --- | --- | --- | --- | --- | --- | --- |
| **BetweenSites** | | Temp. Seasonality → Relative Yield_B_ | -1.40 | -21.2 | (-41.1, -1.27) | 0.037 |
|  |  | Precip. Seasonality → Relative Yield_B_ | 1.68 | 32.7 | (6.75, 58.7) | 0.014 |
|  |  | Temp. Seasonality ↔ Relative Yield_B_ | 0.95 | 0.64 | (0.30, 0.98) | <0.001 |
| **Within Site** | | Inorganic N content → Relative Yield_W_ | 0.27 | 0.69 | (0.02, 1.38) | 0.049 |
|  |  | Soil Health → Relative Yield_W_ | 0.44 | 3.56 | (2.40, 4.71) | <0.0001 |
|  |  | *Soil Health → POXC* | *0.49* | *66.2* | *(47.0, 85.4)* | *<0.0001* |
|  |  | *Soil Health → SOC* | *0.94* | *0.47* | *(0.40, 0.54)* | *<0.0001* |
|  |  | *Soil Health → Soil protein* | *0.75* | *0.73* | *(0.58, 0.88)* | *<0.0001* |
| ^a^ n_B_ and n_W_ indicate sample sizes for the between and within site levels of the model, respectively | | | | | |  |

**Table S8.** Regression coefficients and bootstrapped confidence interval of each relationship in the final N fertilization rate model (n_B_ = 29 sites; n _W_ = 384 samples^a^). Italicized values indicate factor loadings rather than regression coefficients.

| **Level** | **Regression** | **Standardized**  **estimate (β)** | **Unstandardized**  **estimate (B)** | **95% CI**  **(unstandardized)** | **p-value** |
| --- | --- | --- | --- | --- | --- |
| **Between**  **Sites** | MAP → Relative Yield_B_ | -0.35 | -0.02 | (-0.03, 0.00) | 0.086 |
| **Within Site** | Soil Health → Relative Yield_W_ | 0.13 | 0.12 | (-0.01, 0.25) | 0.069 |
|  | Relative fertilization rate → Relative Yield_W_ | 0.74 | 0.75 | (0.65, 0.84) | <0.0001 |
|  | Relative fertilization rate → Soil health | 0.39 | 0.43 | (0.26, 0.59) | <0.0001 |
|  | *Soil Health → POXC* | *0.58* | *0.54* | *(0.42, 0.65)* | *<0.0001* |
|  | *Soil Health → SOC* | *0.79* | *0.73* | *(0.60, 0.86)* | *<0.0001* |
|  | *Soil Health → Soil protein* | *0.64* | *0.59* | *(0.48, 0.70)* | *<0.0001* |
| ^a^ n_B_ and n_W_ indicate sample sizes for the between and within site levels of the model, respectively | | | | | |

**Table S9.** Location, soil classification, and management information for each site.

| **Study Name** | **Site #** | **Location** | **Soil Series** | **Taxonomic description** | **Textural class** | **Tillage** | **Rotation** | **Cover crop** | **Sampling depth (cm)** | **State** |
| --- | --- | --- | --- | --- | --- | --- | --- | --- | --- | --- |
| N Rotation | 1 | 42.93661, -92.57011 | Kenyon | Fine-loamy, mixed, superactive, mesic Typic Hapludolls | loam | conventional-till | Continuous corn (grain), corn-soybean, corn-corn-corn-soybean | None | 0-15 | IA |
| Manure × CC | 2 | 43.30129, -89.34742 | Plano | Fine-silty, mixed, superactive, mesic Typic Argiudolls | silt loam | reduced tillage | Corn (grain) | Barley, none | 0-30 | WI |
|  | 3 | 44.75818, -90.09975 | Withee | Fine-loamy, mixed, superactive, frigid Aquic Glossudalfs | silt loam | conventional-till | Corn (grain) | Barley, none | 0-30 | WI |
|  | 4 | 44.1198, -89.53573 | Plainfield | Mixed, mesic Typic Udipsamments | sand | No-till | Corn (grain) | Barley, none | 0-30 | WI |
|  | 5 | 42.83066, -90.78855 | Fayette | Fine-silty, mixed, superactive, mesic Typic Hapludalfs | silt loam | conventional-till | Corn (grain) | Barley, none | 0-30 | WI |
| Radish CC | 6 | 43.42253, -88.29314 | Theresa | Fine-loamy, mixed, superactive, mesic Typic Hapludalfs | silt loam | no-till | Corn-soybean-wheat | Radish, none | 0-30 | WI |
|  | 7 | 43.79907, -87.91845 | Kewaunee | Fine, mixed, active, mesic Typic Hapludalfs | silt loam | no-till | Corn-soybean-wheat | Radish, none | 0-30 | WI |
|  | 8 | 42.72563, -89.02026 | Plano | Fine-silty, mixed, superactive, mesic Typic Argiudolls | silt loam | no-till | Corn-soybean-wheat | Radish, none | 0-30 | WI |
| Purdue N Trials | 9 | 40.47114, -86.99223 | Chalmers | Fine-silty, mixed, superactive, mesic Typic Endoaquolls | silty clay loam | conventional-till | Corn-soybean | None | 0-20 | IN |
|  | 10 | 40.25335, -85.14803 | Pewamo | Fine, mixed, active, mesic Typic Argiaquolls | clay loam | strip tillage | Corn-soybean | None | 0-20 | IN |
|  | 11 | 39.03314, -85.52582 | Cobbsfork | Fine-silty, mixed, active, mesic Fragic Glossaqualfs | silt loam | no-till | Corn-soybean | None | 0-20 | IN |
|  | 12 | 41.43992, -86.92521 | Tracy | Coarse-loamy, mixed, active, mesic Ultic Hapludalfs | sandy loam | conventional-till | Corn-soybean | None | 0-20 | IN |
|  | 13 | 40.29690, -86.90358 | Toronto | Fine-silty, mixed, superactive, mesic Udollic Epiaqualfs | silt loam | conventional-till | Corn-soybean | None | 0-20 | IN |
|  | 14 | 41.10729, -85.39953 | Morley | Fine, illitic, mesic Oxyaquic Hapludalfs | silty clay loam | no-till | Corn-soybean | None | 0-20 | IN |
| Blevins | 15 | 38.12194, -84.48638 | Maury | Fine, mixed, active, mesic Typic Paleudalfs | silt loam | conventional-till and no-till | Continuous corn | None | 0-20 | KY |
| F05 | 16 | 38.12944, -84.48611 | McAfee -Maury -Huntington | Fine, mixed, active, mesic Mollic Hapludalf - fine, mixed, active mesic Typic Paleudalfs - fine-silty, mixed, active, mesic Fluventic Hapludolls | silty clay loam | no-till | Continuous corn, corn-soybean | None | 0-20 | KY |
| Legume CC | 17 | 43.8065, -87.89767 | Manawa | Fine, mixed, active, mesic Aquollic Hapludalfs | silt loam | conventional-till | Corn-wheat | Barley, clover, none | 0-30 | WI |
|  | 18 | 43.80939, -87.8976 | Kewaunee | Fine, mixed, active, mesic Typic Hapludalfs | silt loam | conventional-till | Corn-wheat | Barley, clover, none | 0-30 | WI |
|  | 19 | 43.80057, -87.91917 | Kewaunee | Fine, mixed, active, mesic Typic Hapludalfs | silt loam | conventional-till | Corn-wheat | Barley, clover, none | 0-30 | WI |
| Ohio N Trials | 20 | 40.56152, -84.28631 | Pewamo | Fine, mixed, active, mesic Typic Argiaquolls | clay loam | conventional-till | Corn-soybean-wheat | Oats-cereal rye | 0-20 | OH |
|  | 21 | 39.95756, -81.90373 | Zanesville | Fine-silty, mixed, active, mesic Oxyaquic Fragiudalfs | silt loam | conventional-till | Corn-soybean | Rye | 0-20 | OH |
|  | 22 | 39.94164, -81.94457 | Zanesville | Fine-silty, mixed, active, mesic Oxyaquic Fragiudalfs | silt loam | conventional-till | Corn-soybean | Cereal rye | 0-20 | OH |
|  | 23 | 40.73274, -83.66592 | Blount | Fine, illitic, mesic Aeric Epiaqualfs | silt loam | no-till | Corn-soybean | Cereal rye | 0-20 | OH |
|  | 24 | 40.32361, -82.32527 | Titusville | Fine-loamy, mixed, active, mesic Aquic Fragiudalfs | silt loam | no-till | Corn-soybean | None | 0-20 | OH |
|  | 25 | 40.40753, -82.68032 | Centerburg | Fine-loamy, mixed, active, mesic Aquic Hapludalfs | silt loam | no-till | Corn-soybean | None | 0-20 | OH |
|  | 26 | 41.29715, -84.70729 | Lenawee | Fine, mixed, semiactive, nonacid, mesic Mollic Epiaquepts | silty clay loam | conventional-till | Corn-soybean | None | 0-20 | OH |
|  | 27 | 39.95722, -82.51416 | Bennington | Fine, illitic, mesic Aeric Epiaqualfs | silt loam | no-till | Corn-soybean | None | 0-20 | OH |
|  | 28 | 41.70961, -83.99495 | Brady | Coarse-loamy, mixed, active, mesic Aquollic Hapludalfs | sandy loam | conventional-till | Corn-soybean | None | 0-20 | OH |
|  | 29 | 41.42986, -84.21012 | Hoytville | Fine, illitic, mesic Mollic Epiaqualfs | clay loam | no-till | Corn-soybean-wheat | Cereal rye, radish | 0-20 | OH |

**References**

1. Bottomley, P. S., Angle, J. S., Weaver, R. W. & Zibilske, L. M. Carbon Mineralization. in *SSSA Book Series* (Soil Science Society of America, 1994).

2. Zibilske, L. Carbon mineralization. *Methods of Soil Analysis: Part 2—Microbiological and Biochemical Properties* 835–863 (1994).

3. Wade, J. *et al.* Sources of Variability that Compromise Mineralizable Carbon as a Soil Health Indicator. *Soil Science Society of America Journal* **82**, 243–252 (2018).

4. Hurisso, T. T. *et al.* Soil Protein as a Rapid Soil Health Indicator of Potentially Available Organic Nitrogen. *Agricultural & Environmental Letters* **3**, (2018).

5. NCR. *Recommended Soil Test Procedures for the North Central Region*. (NCR-13 Soil Testing and Plant Analysis Committee, 2011).

6. Cambardella, C. A. *et al.* Estimation of particulate and total organic matter by weight loss-on-ignition. *Assessment methods for soil carbon* 349–359 (2001).

7. Preacher, K. J., Zhang, G., Kim, C. & Mels, G. Choosing the optimal number of factors in exploratory factor analysis: A model selection perspective. *Multivariate Behavioral Research* **48**, 28–56 (2013).

8. Preacher, K. J. & MacCallum, R. C. Repairing Tom Swift’s electric factor analysis machine. *Understanding statistics: Statistical issues in psychology, education, and the social sciences* **2**, 13–43 (2003).

9. Cattell, R. B. The scree test for the number of factors. *Multivariate behavioral research* **1**, 245–276 (1966).

10. Fabrigar, L. R. & Wegener, D. T. *Exploratory factor analysis*. (Oxford University Press, 2011).

11. Raiche, G. & Magis, D. *Package ‘nFactors’: Parallel analysis and non graphical solutions to the Cattell scree test*. (Version, 2014).

12. Revelle, W. R. psych: Procedures for personality and psychological research. (2017).

13. Matsunaga, M. How to Factor-Analyze Your Data Right: Do’s, Don’ts, and How-To’s. *International journal of psychological research* **3**, 97–110 (2010).

14. Cerrato, M. E. & Blackmer, A. M. Comparison of models for describing; corn yield response to nitrogen fertilizer. *Agronomy Journal* **82**, 138–143 (1990).

15. Poffenbarger, H. J. *et al.* Legacy effects of long-term nitrogen fertilizer application on the fate of nitrogen fertilizer inputs in continuous maize. *Agriculture, ecosystems & environment* **265**, 544–555 (2018).

16. Mamo, M., Malzer, G. L., Mulla, D. J., Huggins, D. R. & Strock, J. Spatial and temporal variation in economically optimum nitrogen rate for corn. *Agronomy Journal* **95**, 958–964 (2003).

17. Dhital, S. & Raun, W. R. Variability in optimum nitrogen rates for maize. *Agronomy Journal* **108**, 2165–2173 (2016).

18. Hu, L. & Bentler, P. M. Cutoff criteria for fit indexes in covariance structure analysis: Conventional criteria versus new alternatives. *Structural equation modeling: a multidisciplinary journal* **6**, 1–55 (1999).

19. Akaike, H. A new look at the statistical model identification. *IEEE Transactions on Automatic Control* **19**, 716–723 (1974).

20. Hsu, H.-Y., Kwok, O., Lin, J. H. & Acosta, S. Detecting misspecified multilevel structural equation models with common fit indices: A Monte Carlo study. *Multivariate Behavioral Research* **50**, 197–215 (2015).

21. Browne, M. W. & Cudeck, R. Alternative ways of assessing model fit. *Sage focus editions* **154**, 136–136 (1993).

22. Efron, B. Better bootstrap confidence intervals. *Journal of the American statistical Association* **82**, 171–185 (1987).

23. Hayes, A. F. Beyond Baron and Kenny: Statistical mediation analysis in the new millennium. *Communication monographs* **76**, 408–420 (2009).

24. Bollen, K. A. & Pearl, J. Eight myths about causality and structural equation models. in *Handbook of causal analysis for social research* 301–328 (Springer, 2013).

25. Magdoff, F. R., Ross, D. & Amadon, J. A Soil Test for Nitrogen Availability to Corn. *Soil Science Society of America Journal* **48**, 1301–1304 (1984).

26. Bundy, L. G. & Malone, E. S. Effect of residual profile nitrate on corn response to applied nitrogen. *Soil Science Society of America Journal* **52**, 1377–1383 (1988).

27. Geisseler, D., Lazicki, P. A. & Scow, K. M. Mineral nitrogen input decreases microbial biomass in soils under grasslands but not annual crops. *Applied soil ecology* **106**, 1–10 (2016).

28. Geisseler, D. & Scow, K. M. Long-term effects of mineral fertilizers on soil microorganisms – A review. *Soil Biology and Biochemistry* **75**, 54–63 (2014).

29. Spackman, J., Fernández, F. G., Coulter, J. A., Kaiser, D. & Paiao, G. Soil texture and precipitation influence optimal time of nitrogen fertilization for corn. *Agronomy Journal* **111**, (2019).

30. Puntel, L. A. *et al.* A systems modeling approach to forecast corn economic optimum nitrogen rate. *Frontiers in plant science* **9**, (2018).

31. Oldfield, E. E., Bradford, M. A. & Wood, S. A. Global meta-analysis of the relationship between soil organic matter and crop yields. *Soil* **5**, 15–32 (2019).

32. Franzluebbers, A. J., Haney, R. L., Honeycutt, C. W., Schomberg, H. H. & Hons, F. M. Flush of Carbon Dioxide Following Rewetting of Dried Soil Relates to Active Organic Pools. *Soil Science Society of America Journal* **64**, 613 (2000).

33. Fine, A. K., van Es, H. M. & Schindelbeck, R. R. Statistics, scoring functions, and regional analysis of a comprehensive soil health database. *Soil Science Society of America Journal* **81**, 589–601 (2017).

34. Mallarino, A. P., Ortiz-Torres, E. & Pringnitz, B. A. A long-term look at crop rotation effects on corn yield and response to nitrogen fertilization. in *Proceedings of the 18th Annual Integrated Crop Management Conference, Iowa State University, Ames* 29–30 (2006).

35. Ruark, M. D. *et al.* Does Cover Crop Radish Supply Nitrogen to Corn? *Agronomy Journal* **110**, 1513–1522 (2018).

36. Moser, M. E. Residual effects of nitrogen fertilization on soil nitrogen pools and corn growth. (2016).

37. Liu, S., Coyne, M. S. & Grove, J. H. Long-term tillage and nitrogen fertilization: Consequences for nitrifier density and activity. *Applied Soil Ecology* **120**, 121–127 (2017).
